# Supplementary material for: Stabilization demands of walking modulate the vestibular contributions to gait
Source: Sci Rep. 2021 Jul 2;11:13736. doi: 10.1038/s41598-021-93037-7 (PMC8253745; doi:10.1038/s41598-021-93037-7)
Supplement: Supplementary file 1 — Supplementary Information. [file 41598_2021_93037_MOESM1_ESM.docx]

**Stabilization demands of walking modulate the vestibular contributions to gait**

Rina M. Magnani^1^, Sjoerd M. Bruijn^2,3^, Jaap H. van Dieën^2^, Patrick A. Forbes^4^ *

^1^Department of Physiotherapy, School of Physical Education and Physical Therapy, State University of Goiás, Goiânia/GO, Brazil

^2^Department of Human Movement Sciences, Faculty of Behavioral and Movement Sciences, Vrije Universiteit Amsterdam, Amsterdam Movement Sciences, Amsterdam, The Netherlands

^3^Institute Brain and Behavior Amsterdam, Amsterdam, The Netherlands

^4^Department of Neuroscience, Erasmus MC, University Medical Center Rotterdam, Rotterdam, The Netherlands

*Corresponding author:

Patrick Forbes
Department of Neuroscience
Erasmus University Medical Centre
P.O. Box 2040
3000 CA, Rotterdam
The Netherlands

[p.forbes@erasmusmc.nl](mailto:p.forbes@erasmusmc.nl)


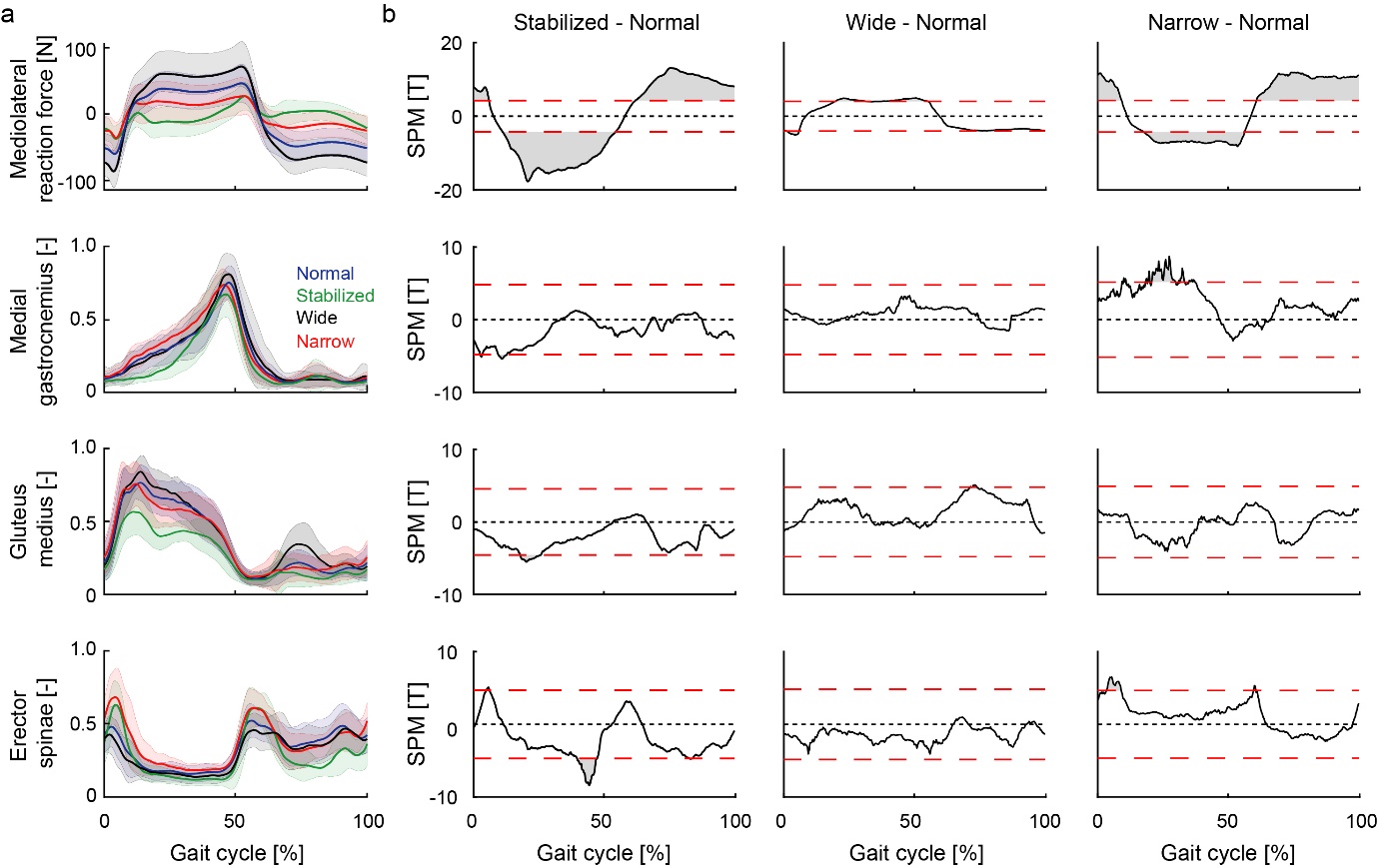


Supplementary Figure S1 – (a) Ground reaction force and electromyographic amplitudes throughout the gait cycle during normal walking (blue line), walking with external lateral stabilization (green line), wide-base walking (black line) and narrow-base walking (red line) (first column) as plotted in Figure 1. (b) Statistical tests of differences (SPM - Statistical Parametric Mapping) in ground reaction force and electromyographic amplitudes between the normal walking condition and walking with external lateral stabilization (second column), wide-base walking (third column) and narrow-base walking (fourth column).
